# Palatal Actinomycosis and Kaposi Sarcoma in an HIV-Infected Subject with Disseminated *Mycobacterium avium-intracellulare* Infection

**DOI:** 10.1155/2012/679728

**Published:** 2012-03-01

**Authors:** Yuria Ablanedo-Terrazas, Christopher E. Ormsby, Gustavo Reyes-Terán

**Affiliations:** Centro de Investigación en Enfermedades Infecciosas, Instituto Nacional de Enfermedades Respiratorias, Mexico City, Mexico

## Abstract

*Actinomyces* and *Mycobacterium avium-intracellulare* are facultative intracellular organisms, members of the bacterial order actinomycetales. Although *Actinomyces* can behave as copathogen when anatomic barriers are compromised, its coinfection with *Mycobacterium avium-intracellulare* has not previously been reported. We present the first reported case of palatal actinomycosis co-infection with disseminated MAC, in an HIV-infected subject with Kaposi sarcoma and diabetes. We discuss the pathogenesis of the complex condition of this subject.

## 1. Introduction

*Actinomyces* are saprophyte bacteria of the human oral cavity and gastrointestinal tract, commonly found in saliva and dental plaque. Under particular circumstances that compromise anatomical barriers, the pathogenic form of *Actinomyces* is enabled to cause actinomycosis, a granulomatous infection of the cervicofacial, thoracic, abdominal, and pelvic regions [1]. The disease is often caused by *A. israellii,* but also by other actinomycetes such as *A. naeslundii*, *A. odontolyticus*, *A. viscous, A. meyeri*, and *A. gerencseriae *[2].

*Actinomyces* and **Mycobacterium avium-intracellulare** complex (MAC) are facultative intracellular organisms, members of the bacterial order actinomycetales. While profound levels of immunosuppression have been associated with disseminated MAC infection in HIV-infected subjects [3], it has not been possible to determine whether HIV infection plays a contributory or a coincidental role in actinomycosis [4]. We present the first reported case of palatal actinomycosis coinfection with disseminated MAC, in an HIV-infected subject with Kaposi sarcoma and diabetes, and provide a discussion of the pathogenesis.

## 2. Case Report

**Figure 1:**
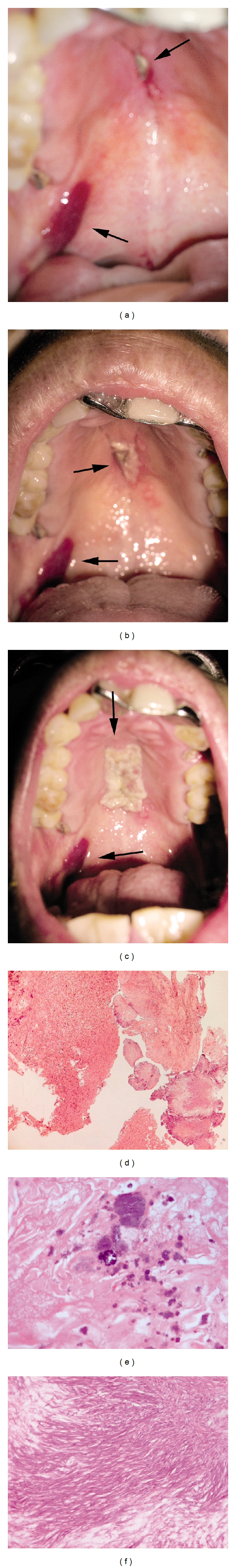
Top row (a) Day 0, biopsy performed: a sessile, whitish lesion of 4-5 mm in diameter is observed in the middle line of the hard palate (upper arrow). There is a violet plaque-like lesion, measuring 1 × 0.8 cm in diameter, compatible with Kaposi's sarcoma on the right side of the soft palate (lower arrow). (b) Day 3: the lesion extended causing erosion of the palatal bone. (c) Day of hospital admission and initiation of treatment (day 7): the palatal lesion extended, measuring 2 × 1 cm in diameter, presenting necrotic tissue, sulphur granules (and erosion of the palatal bone but in absence of nasal cavity fistula. Bottom row. (d) 10x view of the palatal lesion biopsy by hematoxylin eosin staining, showing actinomyces bacteria. (e) 40x magnification of the sulphur granules. (f) 40x magnification of hyphae-like actinomyces strings in the palatal specimen. Digital art disclaimer: for best identification of relevant features, images were adjusted for contrast, brightness, intensity, and saturation, but no brush strokes or similar manipulations were carried out, except for the line art arrows and letters.

A 42-year old-subject presented with 1-week history of small, painless plaque in the middle of the hard palate, which continued growing rapidly during the following days (Figure 1). The patient had lost weight during the previous 2 months and had nocturnal diaphoresis. He denied having fever or chills. He had been diagnosed with type II diabetes mellitus five years earlier and had received an irregular treatment with glibenclamide. Seven months before presentation he had also been diagnosed with HIV infection and he was treated with lopinavir, ritonavir, tenofovir, and emtricitabine. He did not have a history of previous opportunistic infections and CD4+ count was 63 cells/*μ*L. On physical examination he presented normal vital signs. There was a violaceous regular plaque in the right soft palate and a whitish sessile lesion in the middle of the hard palate. There was also a firm preauricular mass measuring 1 cm in diameter on the right side, firmly attached to the parotid gland. The left parotid gland was normal and there was no other palpable lymphadenopathy. No abnormalities were detected in pharynx, larynx, and nasal endoscopy. He presented a purple papule in the abdominal skin. The remainder of the examination was normal. Biopsies of the hard palate and preauricular lymphadenopathy were performed. Pathological examination of the palatal specimen showed sulfur granules and numerous Gram-positive hyphae-like structures indicative of actinomycosis infection. The pathological examination of the lymph node indicated Kaposi's sarcoma, and *Mycobacterium avium- intracellulare* resistant to clarithromycin was cultured from it. Patient received intravenous ampicillin for 2 weeks. As total involution of the hard palate lesion was observed, no surgical debunking was required. He was also treated with clarithromycin plus ethambutol.

## 3. Discussion

We report a rare case of palatal actinomycosis coinfection with disseminated infection by *Mycobacterium avium-intracelulare, *in an HIV-infected subject with diabetes, that presented Kaposi's sarcoma involving the palatal and lymph nodes.

Actinomycosis has not been considered as an opportunistic infection, and an increased incidence in immunosuppressed groups has not been formally demonstrated in any report [4, 5]. HIV infection does not appear to predispose to actinomycosis, as indicated by the low prevalence of the disease in this population [6]. Therefore, it is generally accepted that after mucosal injury, *Actinomyces *is inoculated into submucosal tissues, where it becomes pathogenic by surrounding synergistic bacteria able to create an anaerobic environment. In this particular case, we consider that the palatal Kaposi's sarcoma* caused *the trauma preceding the development of *Actinomyces*. Kaposi's sarcoma is the most prevalent oral neoplasm in HIV-infected patients [7], so rapid identification of coinfecting pathogens in Kaposi's lesions should be performed in order to avoid further complications. In our patient, the palatal plaque was rapidly extending. But it seems likely that additional factors altering homeostatic conditions of the host favored development of actinomycosis. One such factor could be the uncontrolled diabetes as an underlying disease in our patient. Indeed, diabetes has been described as a predisposing factor for infection by *Actinomyces,* and a similar mechanism involving disruption of tissular integrity has been proposed in diabetic patients [8]. Diabetes mellitus is common in our population; the World Health Organization (WHO) estimates that more than 180 million people worldwide have diabetes [9]. By consequence, prompt diagnosis of actinomycosis in this group of patients will lead to a proper antibiotic use in order to eradicate such an infection.

A variety of accompanying bacteria are normally found in cultures from the lesions of actinomycosis; for example, *Actinobacillus actinomycetemcomitans, Eikenella corrodens, *and species of *Fusobacterium, Bacteroides, Capnocytophaga, Staphylococcus, Streptococcus,* and *Enterococcus *have commonly been isolated in various combinations, depending on the site of infection [2]. The exact role of these organisms is still unknown, but it has been speculated that they have a role in reducing local oxygen tension and inducing anaerobiosis inhibition of phagocytes [10] that would in turn allow the anaerobic bacteria to proliferate. **Mycobacterium avium-intracellulare **has not been reported as concomitant bacteria, but is the most common bacteria isolated from Acquired Immunodeficiency Syndrome (AIDS) patients. Individuals infected by **Mycobacterium avium-intracellulare** who have AIDS and/or lymphomas usually develop disseminated MAC (DMAC) infection when they are profoundly immunosuppressed and their CD4+ count falls below 50 cells/*μ*L. Considering that this patient presented disseminated MAC, the possibility that this atypical mycobacteria could have a synergistic role should not be discarded. Previously unsuspected associated copathogens are being described; this could be the case of abdominal coinfection with* M. bovis* and actinomycosis, which has been reported in an individual with AIDS [11].

Confirmation of actinomycosis requires anaerobic culture and the observation of sulphur granules in purulent material or infected tissue [12]. Histological diagnosis of actinomycosis is complicated because many specimens contain only a few granules, and isolation of *Actinomyces in vitro *might be difficult due to strict anaerobic culturing conditions and previous use of antibiotics. By consequence, the medical relevance of actinomycosis might have been underestimated due to difficulties in diagnosing this disease, as a high index of suspicion is also required.

## 4. Conclusion

This case is one of the few case reports of actinomycosis in HIV-infected patient found in the English literature and the only one associated with *Mycobacterium avium-intracellulare* infection and palatal and lymph node disseminated Kaposi sarcoma in a diabetic patient. The indolent course of the rapidly progressive actinomycosis in this patient, not associated with fever, pain, or swelling, could be secondary to his immunocompromised condition. This case in an example of the great variability of pathologies that can be present in an HIV-infected patient and the important role of the otolaryngologist in the evaluation and treatment of this infectious condition.
